# Progress Toward Rubella and Congenital Rubella Syndrome Control and Elimination — Worldwide, 2012–2020

**DOI:** 10.15585/mmwr.mm7106a2

**Published:** 2022-02-11

**Authors:** Laura A. Zimmerman, Jennifer K. Knapp, Sébastien Antoni, Gavin B. Grant, Susan E. Reef

**Affiliations:** ^1^Global Immunization Division, Center for Global Health, CDC; ^2^Department of Immunization, Vaccines, and Biologicals, World Health Organization, Geneva, Switzerland.

Rubella virus is a leading cause of vaccine-preventable birth defects and can cause epidemics. Although rubella virus infection usually produces a mild febrile rash illness in children and adults, infection during pregnancy, especially during the first trimester, can result in miscarriage, fetal death, stillbirth, or an infant born with a constellation of birth defects known as congenital rubella syndrome (CRS). A single dose of rubella-containing vaccine (RCV) can provide lifelong protection against rubella (*1*). The Global Vaccine Action Plan 2011–2020 (GVAP) included a target to achieve elimination of rubella in at least five of the six World Health Organization (WHO) regions* by 2020 (*2*), and WHO recommends capitalizing on the accelerated measles elimination activities as an opportunity to introduce RCV (*1*). This report updates a previous report (*3*) and summarizes global progress toward control and elimination of rubella and CRS from 2012, when accelerated rubella control activities were initiated, through 2020. Among 194 WHO Member States, the number with RCV in their immunization schedules has increased from 132 (68%) in 2012 to 173 (89%) in 2020; 70% of the world’s infants were vaccinated against rubella in 2020. Reported rubella cases declined by 48%, from 94,277 in 2012 to 49,136 in 2019, and decreased further to 10,194 in 2020. Rubella elimination has been verified in 93 (48%) of 194 countries including the entire Region of the Americas (AMR). To increase the equity of protection and make further progress to eliminate rubella, it is important that the 21 countries that have not yet done so should introduce RCV. Likewise, countries that have introduced RCV can achieve and maintain rubella elimination with high vaccination coverage and surveillance for rubella and CRS. Four of six WHO regions have established rubella elimination goals; the two WHO regions that have not yet established an elimination goal (the African [AFR] and Eastern Mediterranean [EMR] regions) have expressed a commitment to rubella elimination and should consider establishing a goal.

## Immunization Activities

The preferred strategy for introducing RCV into national immunization programs is to conduct an initial vaccination campaign targeting the majority of persons who might not have been naturally exposed to rubella, usually children and adolescents aged ≤14 years (*1*), a strategy that has been used to eliminate rubella and CRS in AMR (*4*). WHO recommends that countries that introduce RCV achieve and maintain a minimum coverage of at least 80% with at least 1 dose of RCV delivered through routine services or campaigns (*1*).

Each year, countries report immunization data to WHO and UNICEF using the Joint Reporting Form, which includes information on immunization schedules and the number of vaccine doses administered through routine immunization services and vaccination campaigns.^†^ Because RCV first became available in high-income countries, the World Bank income groupings for 2020 were used to evaluate national income-related disparities.^§^

**FIGURE 1:**
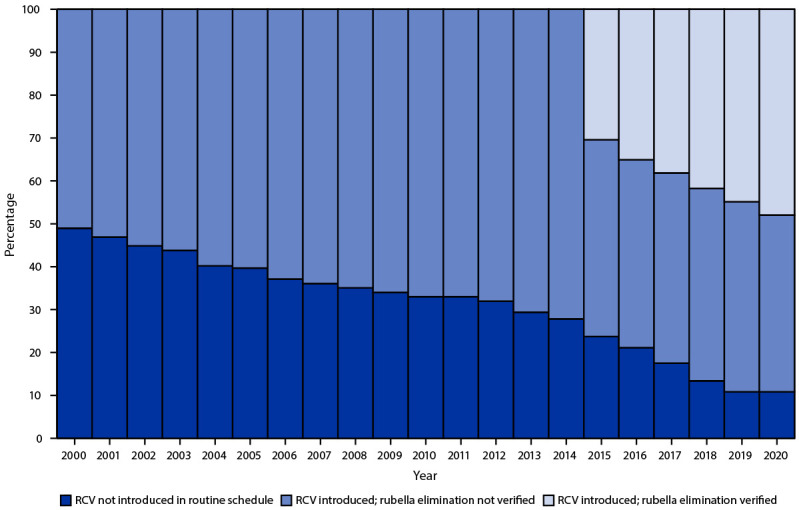
Percentage of countries that have introduced rubella-containing vaccine in the routine immunization schedule and the percentage with verified rubella elimination, by year — worldwide, 2000–2020 **Abbreviation:** RCV = rubella-containing vaccine.

In 2020, RCV had been introduced in 173 (89%) of 194 countries, a 31% increase compared with the 132 (68%) countries that offered RCV in 2012 (Figure 1). All countries in AMR, the European Region (EUR), the South-East Asia Region (SEAR), and the Western Pacific Region (WPR), have introduced RCV. In the two remaining regions, RCV has been introduced in 31 (66%) of 47 countries in AFR, and 16 (76%) of 21 countries in EMR (Table).

**TABLE:** Global progress toward control and elimination of rubella and congenital rubella syndrome, by World Health Organization region — worldwide, 2012, 2019, and 2020

Characteristic	WHO region (no. of countries)						
	AFR (47)	AMR (35)	EMR (21)	EUR (53)	SEAR (11)	WPR (27)	Worldwide (194)
Regional rubella or CRS target	**None**	**Elimination**	**None**	**Elimination**	**Elimination**	**Elimination**	**None**
Countries verified eliminated, no. (%)*							
2012	NA	NA	NA	NA	NA	NA	NA
2019	NA	35 (100)	3 (14)	45 (85)	N/A	4 (15)	87 (45)
2020	NA	35 (100)	3 (14)	49 (92)	2 (18)	4 (15)	93 (48)
**Countries with RCV in schedule, no. (%)**							
2012	3 (6)	35 (100)	14 (67)	53 (100)	5 (45)	22 (81)	132 (68)
2019	31 (66)	35 (100)	16 (76)	53 (100)	11 (100)	27 (100)	173 (89)
2020	31 (66)	35 (100)	16 (76)	53 (100)	11 (100)	27 (100)	173 (89)
**Regional rubella vaccination coverage (%)^†^**							
2012	0	94	38	95	5	86	40
2019	33	87	45	96	93	95	71
2020	36	85	45	94	87	95	70
**Countries reporting rubella cases, no. (%)**							
2012	41 (87)	35 (100)	18 (86)	47 (89)	11 (100)	23 (85)	175 (90)
2019	45 (96)	34 (97)	19 (90)	49 (93)	10 (91)	22 (81)	179 (92)
2020	38 (81)	30 (86)	13 (62)	33 (62)	8 (73)	13 (48)	135 (70)
**Reported rubella cases, no.**							
2012	10,850	15	1,681	30,579	6,877	44,275	94,277
2019	6,027	25	2,603	671	4,537	35,273	49,136
2020	4,883	7	732	92	1,514	2,966	10,194
**Countries reporting CRS cases, no. (%)**							
2012	20 (43)	35 (100)	9 (43)	43 (81)	6 (55)	17 (63)	130 (67)
2019	18 (38)	32 (91)	13 (62)	42 (79)	7 (64)	19 (70)	131 (68)
2020	13 (28)	32 (91)	10 (48)	38 (72)	8 (73)	11 (41)	112 (58)
**Reported CRS cases, no.**							
2012	69	3	20	62	14	134	302
2019	9	0	26	8	358	22	423
2020	28	2	309	2	248	14	603

**Abbreviations:** AFR = African Region; AMR = Region of the Americas; CRS = congenital rubella syndrome; EMR = Eastern Mediterranean Region; EUR = European Region; NA = not available; RCV = rubella-containing vaccine; SEAR = South-East Asia Region; WHO = World Health Organization; WPR = Western Pacific Region.

* Established regional verification commissions verify achievement of elimination in five regions (AMR, EMR, EUR, SEAR, and WPR).  **^†^** Coverage estimates for RCVs are determined by WHO and UNICEF estimates of national immunization coverage.

**FIGURE 2:**
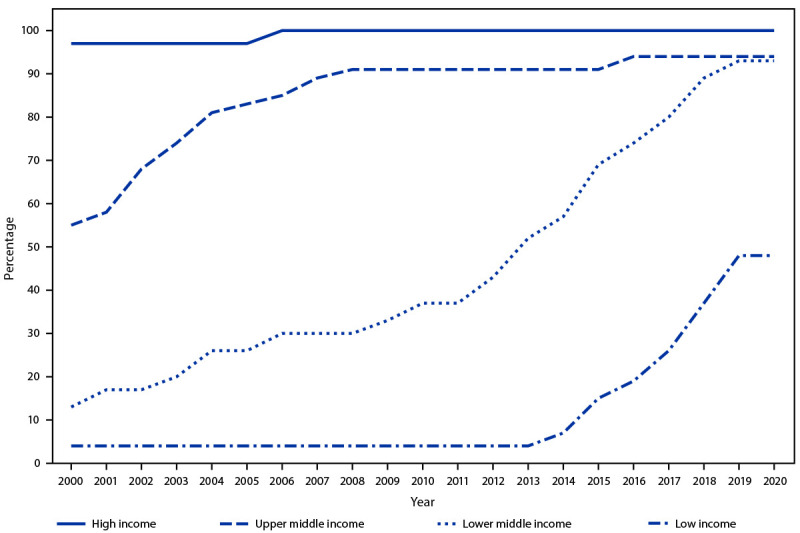
Percentage of countries that have introduced rubella-containing vaccine in the routine schedule, by World Bank income group* and year — worldwide, 2000–2020^†^ * Gross National Income per capita in U.S. dollars in 2020: high income >$12,695; upper middle income = $4,096–$12,695; lower middle income = $1,046–$4,095; and low income ≤$1,045. https://datahelpdesk.worldbank.org/knowledgebase/articles/906519-world-bank-country-and-lending-groups ^†^ In 2020, there were 59 high-income, 54 upper middle-income, 54 lower middle-income, and 27 low-income countries.

The introduction of RCV within income groups has increased over time (Figure 2). In 2012, RCV had been introduced in all 59 high-income countries, 91% of 54 upper middle-income countries, and 43% of 54 lower middle-income countries, but only 4% of 28 low-income countries. By 2020, RCV introduction within income groups increased to 94% of upper middle-income countries, 93% of lower middle-income countries, and 48% of low-income countries.

According to the WHO/UNICEF Estimates of National Immunization Coverage, global infant RCV coverage estimates increased from 40% in 2012 to 70% in 2020, with wide regional variation (range = 36%–95%) (Table). In 2020, rubella vaccination coverage was 26% in low-income counties, 76% in lower middle-income countries and upper middle-income countries combined, and 93% in high-income countries.

## Surveillance Activities and Reported Rubella and CRS Incidence

Rubella and CRS surveillance data are reported through the Joint Reporting Form using standard case definitions (*5*). Rubella and CRS surveillance data complement each other to provide a more complete picture of program progress. Rubella surveillance relies on the measles surveillance system to detect cases because both illnesses cause fever and rash; however, rubella is typically milder than measles, resulting in a lower percentage of persons with rubella seeking health care and a lower percentage of cases being identified. CRS cases are detected through separate surveillance systems, often using a few sentinel sites, which might not be nationally representative (*6*).

In 2020, all 194 countries conducted rubella surveillance, and 193 (99%) had access to standardized quality-controlled laboratory testing through the WHO Global Measles and Rubella Laboratory Network.^¶^ The number of countries reporting rubella cases (including the reporting of zero cases) increased from 175 (90%) in 2012 to 179 (92%) in 2019, but then decreased to 135 (70%) in 2020 during the COVID-19 pandemic. Similarly, the number of countries reporting CRS cases increased from 130 (67%) in 2012 to 131 (68%) in 2019, but then decreased to 112 (58%) in 2020. Compared with the 94,277 rubella cases reported in 2012, case counts declined by 48%, to 49,136 in 2019, with a further decrease to 10,194 in 2020. Reported CRS cases increased from 302 in 2012 to 603 in 2020, primarily because of initiation of CRS surveillance and reporting in several populous countries (Bangladesh, India, Indonesia, and Pakistan) since 2012 and changes in reporting in Pakistan in 2020** (Table). Between 2018 and 2021, 4,588 rubella sequences from 25 countries were reported to the global Rubella Virus Nucleotide Surveillance database^††^; 3,205 (70%) were genotype 1E and 1,382 (30%) were genotype 2B. However, 98% of the sequences were from China and Japan, highlighting the need to enhance global virologic surveillance for rubella.

## Progress Toward Elimination

Progress toward regional goals is measured by the number of countries introducing RCV and the number verified as having eliminated rubella and CRS. The interruption of endemic rubella virus transmission is defined as at least 12 months without ongoing local transmission. When interruption of transmission is sustained for 36 months, an independent regional commission verifies countries as having eliminated rubella (*7*). Data on verification of elimination are available in regional verification commission reports.^§§^^,^^¶¶^^,^***^,^^†††^

During 2019, SEAR advanced its rubella control goal to an elimination goal, joining AMR, EUR, and WPR as regions with rubella and CRS regional elimination goals. Although AFR and EMR have yet to set elimination goals, the regions have expressed a commitment to achieving elimination (*8*). The AMR commission verified that the entire region had eliminated rubella and CRS in 2015; verification commissions in EMR, EUR, SEAR, and WPR assess rubella elimination status on a country-by-country basis. The elimination of endemic rubella has been verified in 93 countries: 35 (100%) in AMR, three (14%) of 21 in EMR, 49 (92%) of 53 in EUR, two (18%) of 11 in SEAR, and four (15%) of 27 in WPR.

## Discussion

Progress toward rubella elimination has accelerated since 2012, and in 2020, rubella elimination had been verified in approximately one half of the countries in the world. The considerable progress made toward elimination has been driven by the establishment of regional WHO rubella elimination goals, an increase in commitment to elimination by countries, and the availability of financial support from global partners for RCV introduction.

Progress is reflected in an increase in the number of countries introducing RCV into national childhood immunization schedules and the coverage achieved. From 2012 to 2020, the number of countries that have introduced RCV increased from 132 to 173, and global coverage increased from 40% to 70%. Although vaccine availability increased, as more low-income countries and lower middle-income countries have introduced RCV, coverage estimates continue to reflect barriers to access in lower-income groups; however, coverage declined only one percentage point from 2019 to 2020 during the COVID-19 pandemic.

Progress has also been reflected in the decline in reported rubella cases, including a 48% decrease during 2012–2019, with a further decrease in 2020. The extent to which rubella transmission declined in 2020 is unclear, however, because fewer reported cases might reflect the impact of COVID-19 mitigation measures or an underreporting of cases in 2020 because of reductions in health care–seeking behavior from patients, health facility availability and reporting, or overall pandemic-related health system disruptions (*9*). The increase in the number of reported CRS cases during 2012–2020 reflects improved surveillance in several populous countries that initiated CRS surveillance after 2012, rather than an increase in rubella among susceptible pregnant women and CRS in their infants. The Measles and Rubella Strategic Framework 2021–2030 outlines potential actions to improve surveillance, including strengthening comprehensive surveillance supported by laboratory networks; promoting training of health workers in early detection, notification and investigation of cases using standardized definitions, tools, and templates for collecting data; and supplementing routine data collection with serosurveys to identify immunity gaps (*8*).

In countries that have not yet introduced RCV, providing policy makers with data on the impact of the investment to introduce RCV can help them determine whether their country should introduce RCV. The decision-making process benefits from 1) evaluation of the impact of RCV introduction on CRS, 2) consideration of the opportunities offered by accelerated measles elimination activities, and 3) evaluation of the long-term sustainability of financing for RCV along with other vaccines (*3*). Countries that had initially introduced RCV in selected populations (usually females only) to control CRS or that introduced RCV without a wide age-range campaign, should identify and address existing immunity gaps to achieve elimination. The Immunization Agenda 2030, the global immunization strategy for 2021–2030, includes rubella in its call for five regions to achieve elimination targets (*10*). Because all six WHO Regions have either established or expressed a commitment to rubella elimination, recommended strategic priorities include improving the collection and use of surveillance data, increasing community demand for and coverage with RCVs, and ensuring the availability of vaccine supplies and laboratory reagents (*8*). Because rubella and measles vaccines are administered as a combined vaccine and the surveillance systems are intricately connected, the progress toward rubella elimination might be a motivating marker of progress toward measles elimination.

The findings in this report are subject to at least two limitations. First, the accuracy and reliability of surveillance and immunization data remain a challenge, limiting the ability to identify immunity gaps, to focus immunization-strengthening activities, and to demonstrate the interruption of rubella virus transmission. Second, the decrease in the number of countries reporting and the effects of the COVID-19 pandemic on the quality of surveillance data limit the ability to monitor progress in 2020.

Considerable progress has been made in control and elimination of rubella and CRS since 2012. By 2020, only 21 (11%) countries have yet to introduce RCV into the immunization schedule, global RCV coverage has increased by 30%, and one region has eliminated rubella and a second region is close. The commitment to elimination by all regions indicates that global rubella elimination is in sight. As the remaining countries introduce RCVs, surveillance and coverage data will become crucial to identifying and closing immunity gaps and maintaining high routine coverage, with periodic campaigns conducted as necessary to achieve and maintain elimination status.

**Summary**

**What is already known about this topic?**

Congenital rubella syndrome, a devastating constellation of birth defects, is caused by rubella infection during pregnancy. Since 2012, rubella-containing vaccine (RCV) introduction efforts have accelerated worldwide, and a 2020 global policy update recommended that introduction efforts use a strategy that leads to elimination.

**What is added by this report?**

By 2020, 173 (89%) of 194 countries had introduced RCVs, and 93 (48%) had been verified as having eliminated rubella transmission. Vaccination introduction equity improved substantially among lower income countries, but vaccination coverage remains a concern.

**What are the implications for public health practice?**

To further progress, it is important the 21 remaining countries introduce rubella vaccine and that all countries enhance vaccination coverage and surveillance to achieve and maintain elimination.

## Footnotes

*
https://www.who.int/countries

†
https://immunizationdata.who.int/pages/coverage/rcv.html?CODE=Global&YEAR=

§ World Bank annually publishes gross national income classification cutoffs per capita in U.S. dollars. The 2022 fiscal year provides classification data for 2020: high income >$12,695; upper middle income = $4,096–$12,695; lower middle income = $1,046–$4,095; and low income ≤$1,045). https://datahelpdesk.worldbank.org/knowledgebase/articles/906519-world-bank-country-and-lending-groups (Accessed January 3, 2022).

¶ São Tomé and Príncipe did not have access to standardized quality-controlled testing by the WHO Measles and Rubella Laboratory Network in 2020.

** Pakistan initiated CRS surveillance in 2018 at four sentinel sites and reported only laboratory-confirmed cases in 2018 (29) and in 2019 (12). In 2020, however, Pakistan added an additional sentinel surveillance site and reported both 22 laboratory-confirmed cases and 279 clinically confirmed cases, for a total of 301 cases in 2020.

††
https://who-gmrln.org/rubens2/

§§
http://www.emro.who.int/media/news/rvc-declared-bahrain-oman-iran-rubella-measles-free.html

¶¶
https://apps.who.int/iris/handle/10665/344160

***
https://apps.who.int/iris/handle/10665/350119

†††
https://www.euro.who.int/en/health-topics/communicable-diseases/measles-and-rubella/activities/regional-verification-commission-for-measles-and-rubella-elimination-rvc/conclusions-of-the-9th-meeting-of-the-european-regional-verification-commission-for-measles-and-rubella-elimination-rvc
